# Whole-Genome Sequencing of a *Mycobacterium orygis* Strain Isolated from Cattle in Chennai, India

**DOI:** 10.1128/MRA.01080-19

**Published:** 2019-10-03

**Authors:** Ahmed Kabir Refaya, Narender Kumar, Dhinakar Raj, Maroudam Veerasamy, Subramanyam Balaji, Sivakumar Shanmugam, Ananthi Rajendran, Srikanth Prasad Tripathy, Soumya Swaminathan, Sharon J. Peacock, Kannan Palaniyandi

**Affiliations:** a ICMR-National Institute for Research in Tuberculosis, Chennai, India; b Department of Medicine, University of Cambridge, Cambridge, United Kingdom; c Translational Research Platform for Veterinary Biologicals (TRPVB), Tamil Nadu Veterinary and Animal Sciences University, Chennai, India; d World Health Organization, Geneva, Switzerland; University of Rochester School of Medicine and Dentistry

## Abstract

Here, we report the isolation of Mycobacterium orygis from dairy cattle in Chennai, India. Spoligotyping assigned the isolate to spoligotype 587 (ST587), which belongs to M. orygis. This species was confirmed as *M. orygis* using whole-genome sequencing.

## ANNOUNCEMENT

Characterization of *Mycobacterium orygis* as a subspecies of the Mycobacterium tuberculosis complex (MTBC) that can cause clinical features of tuberculosis in animals and humans was first published by Van Ingen et al. in 2012 (1). Several studies have since reported the isolation of *M. orygis* from captive wild animals (2) and from dairy cattle and captured monkeys (3). A putative case of human-to-cattle transmission of *M. orygis* in New Zealand was reported, the source of which was traced back to India based on epidemiological analysis (4). *M. orygis* is suggested to be endemic in Southeast Asia, including in India, Pakistan, and Nepal (4, 5). Here, we report the isolation of *M. orygis* from cattle from India.

A postmortem examination performed on comparative intradermal test (CIT)-positive cattle from a farm in Chennai, India, revealed a macroscopic appearance consistent with severe tuberculosis of the lungs. Tissue samples from the lungs were homogenized, decontaminated with 5% sulfuric acid in phosphate-buffered saline (PBS), filtered with sterile muslin cloth (6), and inoculated onto Lowenstein-Jensen (LJ) slants and mycobacterial growth indicator tubes (MGIT). Positive mycobacterial growth on MGIT and LJ slants was confirmed by Ziehl-Neelsen (ZN) staining, and the MTBC was confirmed by immunochromatographic testing (ICT) (7). Mycobacterial colonies on LJ slants were suspended in Tris-EDTA (TE) buffer, and genomic DNA was isolated by the cetyltrimethylammonium bromide (CTAB)-NaCl method (8). Spoligotyping was performed as previously described (9), and the spoligotype pattern was compared against those in the SpolDB4 database (10). The genomic DNA was checked for quality by measuring the *A*_260/280_ ratio using the NanoDrop method and was quantified using Qubit. The sequencing library was prepared using a TruSeq Nano DNA LT library prep kit as per the manufacturer’s protocol. The quality of the library was checked using an Agilent 2200 tape station. Whole-genome paired-end sequencing was carried out on an Illumina HiSeq 2500 instrument, generating 20,510,576 read pairs of 150 bp. Raw reads were filtered using Trimmomatic v0.36 (quality value, >20; minimum length, >60 bp) (11). Filtered reads were aligned to the reference genome of H37Rv (GenBank accession number NC_000962) using Burrows-Wheeler Aligner (BWA) v0.7.12 (12) with default parameters, and alignments were corrected using GATKv3.5 (13). Variants were called using SAMtools v1.3.1 (14) and bcftools v1.3.1 with default parameters. Variants with a quality of >50, a mapping quality of >30, and a depth of at least 5 with at least one read in either direction were filtered for analysis. RD-Analyzer v1.0 (default parameters) was used to detect regions of difference (RDs; regions of the MTBC genome whose presence or absence is lineage specific and that are widely used as classification markers) (15). Filtered reads were also assembled with SPAdes v3.11.0 with autocorrection (16).

Comparison of the spoligotype profile with those in the SpolDB4 database classified the isolate as spoligotype 587 (ST587), which belongs to *M. orygis* (1). RD-Analyzer results confirmed the absence of RD7, RD8, RD9, and RD10 and the presence of RD1 and RD4 (1). The assembled genome (size, 4,293,394 bp) consisted of 107 contigs with an *N*_50_ value of 99,756 bp and a G+C content of 65.59%. Analysis of identified variants revealed a previously reported T-to-G mutation in the 38th codon of Rv2042c and a C-to-T mutation at the 321st codon in the gene *PPE55* (3). These genomic features, along with the spoligotyping results, confirmed the strain to be *M. orygis.* This genome sequence will contribute to our understanding of the genomic characteristics of this species and will facilitate further comparative genomic studies.

### Data availability.

This whole-genome shotgun project has been submitted to NCBI under BioProject identifier PRJNA545406. The raw reads and the assembled contigs have been submitted under accession numbers SRR9157804 and VDER00000000, respectively. The version of the assembled contigs described in this paper is the first version, VDER01000000.
